# The Effect of Cigarette Smoking during Pregnancy on Endocrine Pancreatic Function and Fetal Growth: A Pilot Study

**DOI:** 10.3389/fpubh.2017.00314

**Published:** 2017-11-21

**Authors:** Fatima Lockhart, Anthony Liu, Bernard Linton Champion, Michael John Peek, Ralph Kay Heinrich Nanan, Alison Sally Poulton

**Affiliations:** ^1^Women and Children’s Health, Nepean Hospital, Penrith, NSW, Australia; ^2^Charles Perkins Centre Nepean, University of Sydney, Sydney, NSW, Australia; ^3^ANU Medical School, Australian National University, Canberra, ACT, Australia

**Keywords:** prenatal cigarette smoke exposure, prenatal nicotine exposure, endocrine pancreatic function, birthweight, insulin resistance, serotonin

## Abstract

**Introduction:**

Cigarette smoking in pregnancy is a common cause of fetal growth restriction. We aimed to investigate endocrine pancreatic function of mother–infant dyads in relation to cigarette smoking, as a possible mechanism for the poor fetal growth.

**Methods:**

Prospective study of smoking mothers (10 cigarettes or more per day, self-reported to the midwife) and non-smoker control mothers during their first pregnancy. Insulin, glucose, C-peptide, HbA1C, fructosamine, prolactin, serotonin, and cortisol were measured in maternal blood at 24–26 weeks and in umbilical cord blood at birth. Cotinine was also measured in cord blood.

**Results:**

Of 37 smokers and 36 non-smokers recruited, cord blood was obtainable from 38 babies (19 in each group). *In utero* cigarette exposure was associated with lower birthweight (3,035 ± 490 versus 3,405 ± 598 g, *p* = 0.005), with linear modeling of the smoking cohort showing a 41 g reduction for every increase of one cigarette smoked per day (95% CI −71 to −11 g, *p* = 0.010). There were no differences between groups in indices of maternal or perinatal endocrine pancreatic dysfunction. Heavier smoking independently correlated with higher maternal fasting levels of glucose (*p* = 0.044) and C-peptide (*p* = 0.011). We did not observe any significant associations between the daily number of cigarettes and any of the cord blood parameters. We also looked for differences between cohorts based on infant gender. Serotonin levels were higher in smoking mothers with male fetuses (*p* = 0.01 to *p* = 0.004).

**Conclusion:**

Endocrine pancreatic dysfunction does not appear to be a major contributing factor to nicotine-associated fetal growth restriction. The higher serotonin levels in smoking mothers carrying male infants is of uncertain significance but could be a manifestation of gender differences in susceptibility to the long-term effects of cigarette smoking.

## Introduction

Cigarette smoking is a common cause of fetal growth restriction (1–3). There are a number possible of mechanisms, including nicotine-induced placental vasoconstriction (4) and the effects of carbon monoxide on mitochondrial function (5). We planned to investigate a possible adverse effect on endocrine pancreatic function (EPF).

During pregnancy, the maternal pancreatic beta cells increase in number and in their glucose sensitivity in response to higher levels of prolactin and lactogen, resulting in increased insulin secretion (6). The glucose-lowering effect of the higher insulin levels is balanced by reduced insulin sensitivity of maternal cells, making glucose more available to the fetus and enhancing fetal growth. The higher insulin concentration also increases the appetite and promotes transport of glucose across the placenta (7). The positive effects of prolactin and lactogen on beta cell mass might be mediated by elevations in pancreatic serotonin, detectable by a rise in circulating serotonin in mid-pregnancy (8).

Research on rats has shown that nicotine damages the fetal pancreatic mitochondria leading to beta cell dysfunction and apoptosis. This causes impaired glucose tolerance in the offspring, which may be irreversible if nicotine exposure continues during lactation (9). Inadequate insulin production in the fetus would impair glucose uptake and utilization, which could be a contributary mechanism for the poor fetal growth. An adverse effect of maternal smoking may persist into childhood; children exposed to cigarette smoke *in utero* tend to have a higher body mass index (BMI), which is not totally attributable to their lower birthweight (10), and there are also gender differences in the childhood growth patterns, with greater weight gain in boys (11). Cigarette smoking also predisposes to gestational diabetes (12).

The aim of this prospective study was to investigate the effects of cigarette smoking in pregnancy. We aimed to compare EPF of smoking and non-smoking mothers in mid pregnancy and to compare the growth parameters and EPF of their infants at birth in relation to cigarette smoke exposure. We considered that the fasting glucose, insulin, and c-peptide would give an indication of the level of glucose and insulin tolerance and the differences in fasting and post-prandial glucose, insulin, and c-peptide would indicate the EPF responsiveness to an immediate glucose load. The levels of fructosamine and hemoglobin A_1_C would give a further indirect measure of EPF. We measured prolactin and serotonin, which promote or mediate beta cell proliferation, to exclude low levels of these hormones as a cause of low insulin levels.

## Materials and Methods

The study was carried out in a tertiary university hospital in a suburb of Sydney in NSW, Australia. Primiparous non-obese women (BMI, 18–30 kg/m^2^), who either smoked at least 10 cigarettes per day (self-reported to the midwife) or had never smoked, were identified from the obstetric database of the hospital maternity unit. This database includes the woman’s age, BMI, expected date of delivery (EDD), and number of cigarettes smoked per day, recorded at the midwifery booking visit. The baby’s birthweight, length, head circumference, and gestational age at birth are subsequently recorded on the database at birth by the midwife attending the delivery. The EDD and gestational age at birth were confirmed by one of the researchers (Fatima Lockhart) from data of early ultrasounds. Eligible women were approached by Fatima Lockhart at their next antenatal visit and recruited into the study after informed consent; only one woman declined participation. The data collection period was from 2011 to 2012. After experiencing some difficulties with cord blood collection at delivery, additional participants were recruited in 2013. Participation involved additional blood taken for EPF with the 2-h glucose tolerance test (75 g oral glucose load administered after an overnight fast) at 24–26 weeks gestation and EPF studies on umbilical cord blood at delivery. Blood collection for the glucose tolerance test was by laboratory staff at the antenatal clinic. The cord blood was usually collected by Fatima Lockhart, or when she was unavailable the midwife would collect the cord blood. In some cases (usually in the babies who were small for gestational age), it was not possible to draw sufficient, if any, blood from the placental vessels after delivery. Sometimes in babies who were born by cesarean section, cord blood collection was missed. Women who took other recreational drugs or who had significant comorbidities were excluded. We recruited and collected maternal blood samples from 37 smokers and 36 controls and obtained cord blood from 19 and 19, respectively. All subjects gave written informed consent in accordance with the Declaration of Helsinki. The study had ethical approval from the Nepean Blue Mountains Human Research Ethics Committee (HREC/11/NEPEAN/27).

HbA1C, fructosamine, prolactin, serotonin, and cortisol were analyzed on the fasting blood samples; insulin, glucose, and C-peptide were analyzed on fasted and 2-h samples. The same parameters and cotinine were measured on umbilical cord blood. Serotonin was measured in serum at SydPath (St. Vincent’s Hospital, Sydney, NSW, Australia) using an in-house high performance liquid chromatography method with electron capture detection. By use of serum as the sample, the assay measures platelet serotonin in addition to any extracellular circulating serotonin. Serum cotinine was measured by immunoassay using the Nicotine Metabolite assay on an Immulite 1000 analyser (Siemens Diagnostics, Australia). The other analyses used the clinical assays that were available from our hospital laboratory. Maternal beta cell function was evaluated on fasted samples using the homeostasis model assessment (HOMA) (13). This included HOMA-B (index of beta cell function based on the ratio of fasting insulin to fasting glucose) HOMA-IR (index of fasting insulin resistance) and HOMA-S (index of insulin sensitivity). These are standardized in relation to the basal (fasted) levels of insulin and glucose expected for a healthy, normal weight person aged less than 35 years.

This was a pilot study and was, therefore, carried out with a view to a larger study if positive results were obtained. We calculated that assuming a SD for fasting glucose concentration of 0.7 mmol/L, a sample size of 20 per group would be able to show a difference between groups of 0.63 mmol/L with 80% confidence at the 5% level of significance. Between group comparisons used independent samples *t*-tests. Correlates of the daily dose of nicotine were assessed using the Pearson correlation. Multiple logistic regression was used to determine the relationship between the various indices of EPF controlling for relevant confounders. Among the smoking mothers, univariate analysis was used to estimate the effect of the daily dose of nicotine on infant birthweight. All analyses were 2-tailed and results were assumed to be statistically significant if the *p*-value was less than 0.05. SAS version 9.3 was used for the analysis.

## Results

A total of 73 women (37 smokers, 36 controls) were recruited and had blood taken for EPF. Cord blood was unobtainable from 35 neonates. Eighteen (51%) of these were nicotine exposed, with a higher mean daily cigarette usage than those with cord blood samples (17 ± 5 versus 11 ± 3, *p* < 0.001). The infants without cord blood were of lower gestational age (38.3 ± 2.1 versus 40.3 ± 2.8 weeks, *p* = 0.001) and birthweight (2,998 ± 672 versus 3,419 ± 370 g, *p* = 0.001).

Women who smoked were younger and had a higher BMI than controls (Table 1) and their infants had lower birthweights (3,035 ± 490 versus 3,405 ± 598 g, *p* = 0.005) with a significant dose effect: linear modeling of the nicotine exposed cohort demonstrated that every increase of one cigarette per day was associated with a 41 g reduction in birthweight (95% CI −71 to −11 g, *p* = 0.010). Although women who smoked had significantly higher c-peptide and borderline elevation of serotonin compared to controls, these differences were not significant after controlling for age and BMI. These differences were not observed in the cord blood samples. There was no significant relationship between smoking and fasting insulin or HOMA index. There were no other significant differences between groups and no stillbirths, neonatal deaths, or intensive care admissions.

**Table 1:** Comparison of cigarette smoke exposed and non-exposed mothers and their infants.

	Smokers mean ± SD	Controls mean ± SD	*p-*Value (*t*-test)			
Number	37 (18 male infants, 49%)	36 (16 male infants, 44%)				

Age (years)	22.78 ± 5.21	27.10 ± 5.30	0.001	**LS means controlling for maternal age and BMI**		
BMI (kg/m^2^)	24.97 ± 3.67	22.86 ± 3.25	0.011			
				
				**Smokers**	**Controls**	***p-*Value**

Birthweight (g)	3,035 ± 490	3,405 ± 598	0.005	3,010	3,430	0.007
				3,005^a^	3,435^a^	0.002
Length (cm)	49.3 ± 2.9	50.3 ± 2.8	0.14	49.2	50.5	0.07
Head circumference (cm)	33.7 ± 1.7	34.1 ± 1.8	0.32	33.7	34.1	0.41
Gestational age (weeks)	39.5 ± 2.6	39.2 ± 2.8	0.60	39.4	39.3	0.91
**Maternal blood (24–26 weeks)**						
Glucose (mmol/L) F	4.42 ± 0.60	4.23 ± 0.37	0.11	4.42	4.24	0.16
2 h	5.53 ± 1.33	6.05 ± 1.36	0.11	5.70	5.87	0.64
Insulin (μU/L) F	13.5 ± 15.6	9.9 ± 20.2	0.40	13.3	10.0	0.50
2 h	55.2 ± 46.7	64.4 ± 65.1	0.50	55.2	64.3	0.57
C-peptide (nmol/L) F	1.02 ± 0.85	0.64 ± 0.28	0.017	0.93	0.73	0.19
2 h	3.39 ± 1.40	3.61 ± 1.79	0.56	3.41	3.60	0.67
HbA1C (%)	5.00 ± 0.58	5.01 ± 0.34	0.96	4.99	5.02	0.81
Fructosamine (μmol/L)	193 ± 18	201 ± 20	0.06	196	198	0.68
Cortisol (nmol/L)	747 ± 165	681 ± 171	0.10	742	686	0.23
Prolactin (mIU/L)	3,928 ± 2,609	4,660 ± 2,073	0.20	3,966	4,622	0.32
Serotonin (nmol/L)	740 ± 375	592 ± 239	0.05	731	602	0.14
Homeostasis model assessment (HOMA) B	29.6 ± 25.1	32.4 ± 51.3	0.29	27.5	34.5	0.53
HOMA IR	0.292 ± 0.399	0.192 ± 0.402	0.14	0.292	0.193	0.36
HOMA S	12.5 ± 15.7	18.7 ± 19.7	0.14	15.3	15.9	0.90
**Umbilical cord blood at birth**						
Number^b^	11–19	15–19				
Glucose (mmol/L)	4.31 ± 1.06	4.48 ± 1.00	0.61	4.36	4.43	0.86
Insulin (μU/L)	3.82 ± 5.60	3.81 ± 2.17	0.99	4.29	3.32	0.57
C-peptide (nmol/L)	0.28 ± 0.15	0.30 ± 0.10	0.70	0.294	0.285	0.83
Fructosamine (μmol/L)	209 ± 30	206 ± 22	0.78	205	209	0.67
Cortisol (nmol/L)	406 ± 355	462 ± 274	0.59	416	452	0.75
Prolactin (mIU/L)	10,097 ± 3,528	8,639 ± 4,841	0.30	9,700	9,090	0.71
Serotonin (nmol/L)	216 ± 87	180 ± 79	0.21	209	187	0.47
Cotinine (μg/L)	24.5 ± 26.7	All samples <8 μg/L	0.00			
	*n* = 11	*n* = 15				

*^a^Controlling for gestational age*.

*^b^Some results missing due to insufficient sample*.

*F, fasted overnight; 2 h= 2 hours following 75 g oral glucose load; BMI, body mass index; LS means, least squares means controlling for confounders*.

**Figure 1:**
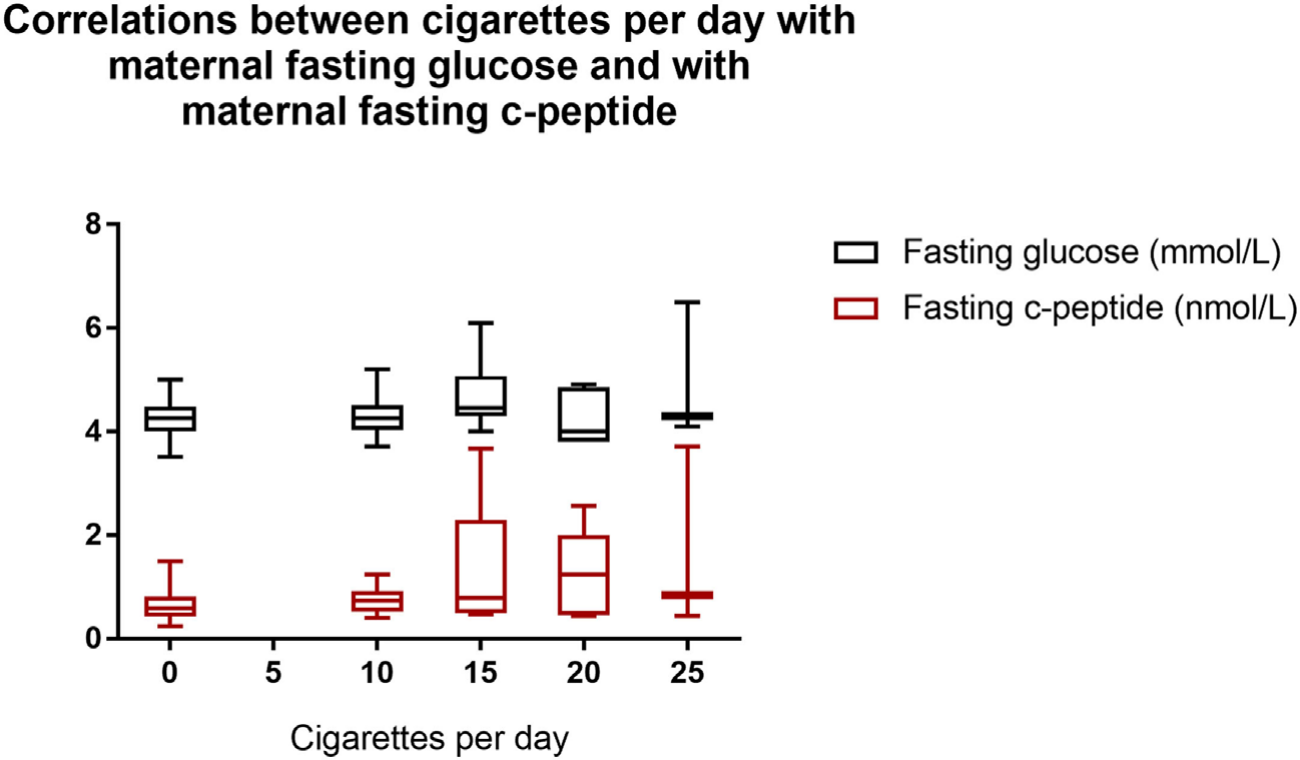
Correlations between cigarettes per day with maternal fasting glucose and with maternal fasting c-peptide. Cigarettes per day with fasting glucose: *r* = 0.27, *p* = 0.022. Cigarettes per day with fasting c-peptide: *r* = 0.40, *p* < 0.001. Box shows interquartile range and error bars indicate full range.

Correlates of the dose of nicotine, using the reported number of cigarettes smoked per day, were sought by combining data from both groups. The number of cigarettes smoked per day correlated with the maternal fasting glucose (*r* = 0.27, *p* = 0.022) and c-peptide (*r* = 0.40, *p* < 0.001) (Figure 1); correlations with serotonin and fructosamine were at borderline significance (*r* = 0.24, *p* = 0.044 and *r* = 0.23, *p* = 0.054, respectively). After controlling for age and BMI, the number of cigarettes smoked per day correlated significantly only with the levels of maternal fasting glucose (*p* = 0.044) and fasting c-peptide (*p* = 0.011). We did not observe any significant associations between the daily number of cigarettes and any of the cord blood EPF parameters. Elevated cotinine levels were found in 8 of 11 samples analyzed from cord blood from infants of mothers who smoked (24.5 ± 26.7 μg/L, *n* = 11) but none of the non-smoking controls (*n* = 15). Among the smokers, the cotinine levels did not correlate with any indicators of pancreatic function, or the reported number of cigarettes smoked per day, or with the birthweight.

**Figure 2:**
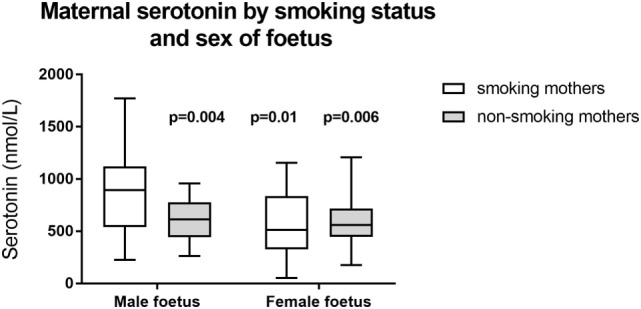
Maternal serotonin by smoking status and sex of fetus. *p*-Values refer to comparison with smoking mothers carrying a male fetus. Other between group comparisons: *p* = 0.79–0.98. Box shows interquartile range and error bars indicate full range.

We also looked for differences between cohorts based on infant gender: we found significant differences only in the maternal serotonin levels. Serotonin levels were significantly higher in smoking mothers with male fetuses: male fetus smoker (*n* = 18), 889 ± 390 nmol/L; male fetus non-smoker (*n* = 14), 606 ± 208 nmol/L (*p* = 0.01 compared to male fetus smoker); female fetus smoker (*n* = 17), 582 ± 316 nmol/L (*p* = 0.004 compared to male fetus smoker); female fetus non-smoker (*n* = 19), 608 ± 243 nmol/L (*p* = 0.006 compared to male fetus smoker). These data are illustrated in Figure 2.

## Discussion

The infants of smoking mothers had lower weight at birth, with a significant dose effect in terms of the number of cigarettes reportedly smoked per day. Despite this evidence of clinically meaningful antenatal exposure to nicotine, we found no significant differences between groups in biochemical indices of EPF, either in the exposed mothers or their infants. However, the significant correlation between the number of cigarettes smoked per day and the fasting levels of glucose and C-peptide is consistent with a nicotine-related reduction in maternal insulin sensitivity.

We could find no other studies investigating the effect of cigarette smoking on EPF in mother–infant dyads. Our finding of measurable levels of cotinine only in the umbilical cord blood of mothers who smoked supports the value of self-reporting of cigarette use during pregnancy (14). However, the actual concentration of cotinine did not show any significant correlation with either the birthweight or the number of cigarettes reportedly smoked per day. This suggests that the umbilical cord cotinine did not accurately reflect the level of the nicotine exposure during pregnancy. This might be due to a change in the smoking pattern around the time of birth.

Strengths of this study include the strict inclusion criteria using only first pregnancies and comparing heavy smokers with women who had never smoked. The main weaknesses of the study were the small numbers and that cord blood was not obtained in about half of the patients recruited. These included heavier smokers with smaller infants, who might have been more likely to show an adverse effect on EPF.

The finding of significantly higher levels of serotonin in smoking mothers with male fetuses was unexpected. However, factors associated with the intrauterine environment may lead to different outcomes depending on the sex of the fetus. Badon et al. found that healthier lifestyle scores in early pregnancy (included in this was cigarette smoking) was associated with greater birthweight for boys but lower birthweight for girls (15). Ayonrinde et al. found an association between maternal smoking and non-alcoholic fatty liver disease among teenaged girls but not boys (16).

There is some evidence that the effects of prenatal nicotine exposure may be worse in male offspring. An association between prenatal nicotine exposure and higher childhood BMI has been reported to be more significant in boys (11). In rats, only male offspring of nicotine-exposed mothers had elevated levels of circulating triglycerides (17). It is tempting to speculate that the apparently normal pancreatic function that we observed may have been sustained by higher serotonin levels in smoking mothers carrying sons.

The difficulty in collecting cord blood on infants likely to be at highest risk for pancreatic damage secondary to cigarette smoking could be overcome by collecting blood from neonates. However, this might be difficult to justify for an exploratory study and experimental work on animals might be more appropriate. A non-invasive alternative would be to measure serotonin in the urine of exposed and unexposed neonates, which might provide indirect evidence of differences in EPF.

In conclusion, we found no significant correlation between indices of EPF and smoking related fetal growth impairment. The higher serotonin levels in smoking mothers carrying male infants is of uncertain significance but could be a manifestation of the recognized gender differences in susceptibility to the long-term effects of prenatal nicotine exposure. This finding needs further research.

## Implications

Despite a significant adverse effect of smoking on birthweight, this small pilot study did not indicate any significant effect of smoking on EPF. This implies that endocrine pancreatic dysfunction is not the main mechanism for the fetal growth restriction associated with cigarette smoking. However, the finding of higher serotonin levels in the smoking mothers carrying sons merits further investigation.

## Ethics Statement

All subjects gave written informed consent in accordance with the Declaration of Helsinki. The protocol was approved by the the Nepean Blue Mountains Human Research Ethics Committee (HREC/11/NEPEAN/27).

## Author Contributions

Conception and design of the study: AP, RN, MP, AL. Analysis and interpretation of data: AP, RN, FP, BC. Drafting the article: FL, AP. Revising the article for critical intellectual content: RN, AL, BC, MP. Approval of the final version: FL, AP, RN, FP, BC, AL.

## Conflict of Interest Statement

AP reports personal fees and non-financial support from Shire, outside the submitted work and shares in GSK. BC reports personal fees from Novartis, MSD and Astra Zeneca and non-financial support from Novartis, outside the submitted work. RN, MP, AL, and FL report no conflicts of interest.
